# Calcifying Epithelial Odontogenic Cysts of the Anterior Maxilla: Report of Two Cases

**DOI:** 10.7759/cureus.65392

**Published:** 2024-07-25

**Authors:** Sonalee J Shah, Sarita Tandon, Chandani Ratnani, Indu Sonwani, Jayanti Bishal

**Affiliations:** 1 Oral Pathology, Government Dental College, Raipur, IND

**Keywords:** gorlin cyst, dental lamina, odontogenic pathology, ghost cells, calcifying odontogenic cyst

## Abstract

This article is a discussion of two cases of young adults with lesions in similar locations in the anterior maxilla, i.e., the canine-to-canine region, similar history, and comparable radiology. Both cases were histologically diagnosed as calcifying odontogenic cysts.

Case 1 was a male aged 28 years with diffuse, firm left malar area facial swelling with pain in associated teeth for a month. Intraorally, he had a gingivo-vestibular swelling also extending palatally in the anterior left maxillary region extending from the distal surface of the left maxillary central incisor to the mesial surface of the left maxillary canine. The overlying mucosa was normal in appearance. The radiograph showed a large unilocular radiolucency in the affected region. The lesion was excised followed by curettage and primary closure.

Case 2 was a female aged 25 years with a lumpy mass and pain in associated teeth since one year in the left canine-premolar region with an external swelling in the left ala of the nose region that extended superiorly to the zygomatic arch. The color of the skin as well as the intraoral mucosa was normal, and an orthopantomogram (OPG)revealed a unilocular radiolucency in the left maxillary canine-premolar region with resorption of premolar roots. Treatment included surgical enucleation and bone curettage.

Both cases have been in follow-up for about a year and have shown non-incidental healing.

## Introduction

An active interaction of odontogenic mesenchyme and epithelium is essential to the production of dentin and enamel in the tooth germ genesis. These odontogenic tissues are prone to experience cystic and/or tumorous alterations prior to hard tissue formation, that result in the development of odontogenic diseases, particularly, when the odontogenic tissues dedifferentiate or when remnant/residual cells are activated. It is believed that either the remaining dental lamina or the diminished enamel epithelium surrounding the unerupted tooth crown serves as the parent source for the initiation of calcifying odontogenic cysts (COCs) [1].

The initiation of COC is, therefore, associated with the occurrence of odontogenic leftovers in the bone or gingival part and can manifest as either an extraosseous gingival (peripheral, approximately 20%) lesion or an intraosseous (central), which is the more prevalent kind of pathology. The intraosseous type of COC is typically asymptomatic and frequently accompanies an impacted tooth. In the mouth, it has a propensity for developing in the canine/premolar region of the jaws, thus, suggesting a preference for the anterior portion of both jaws. Calcifying odontogenic cysts (COC) have a wide range of histologic heterogeneity as well as inconsistent clinical behavior. Normally, the lining epithelium is thin and prone to separation, and the cyst epithelium tends to be focally thickened by virtue of the presence of ghost cells and keratinized epithelial cells in the cyst lining [1,2].

About 2% of all odontogenic pathologies are calcifying odontogenic cysts (COCs) and have an incidence of 0.3% to 0.8% of all odontogenic cysts. Hence, it is an uncommon benign odontogenic cyst. At first, it was thought of as the oral counterpart of Malherbe's cutaneous calcifying epithelioma, but he subsequently classified it as COC [3].

The literature in the fields of medicine and dentistry frequently claims that COC was identified as a unique clinicopathological entity for the first time in 1962. However, the first report on CEOC (calcifying epithelial odontogenic cyst) was published five decades earlier. It was later outlined as a pathology with elaboration of its features by the efforts of various researchers [3,4].

According to Mullvhill et al., despite the lesion's widespread perception of being a benign odontogenic cyst, the COC was reclassified as a calcifying cystic odontogenic tumour, under the tumour classification in the WHO Category of 2005. The neoplastic variant of the tumour was still referred to as a dentinogenic ghost cell tumour. The *WHO Classification of Head and Neck Tumors, Fourth Edition*, which was just published, has restored COC to the category of an odontogenic cyst and discards the preceding terms [4].

As many as 75-77% of cases of COC tend to show resorption of teeth roots in its vicinity, and COCs are frequently affiliated with an impacted tooth, as already mentioned. Consequently, the main clinical manifestations frequently vary from teeth loosening to enlargement of the focal affected area [4].

The diagnosis of COC is important as very often it occurs as an asymptomatic intraosseous pathology with no pathognomic clinical or radiological signs and is often seen to be associated with other odontogenic pathologies. Therefore, it is usually diagnosed histopathologically only and the diagnosis is important to avoid radical surgical treatment as this cyst usually has an excellent prognosis with very few recurrences. Malignant transformation of COC is even less likely or rarer.

The objective of the current publication is to discuss two cases of COC with an update and comparison of their clinical behavior, radiographical, histopathological, differential diagnosis, treatment, and prognostic findings in order to reassert the likelihood of COC behaving as a locally aggressive lesion, categorized under benign odontogenic ghost cell lesions (BOGCL) but with a possibility of malignant transformation. 

## Case presentation

Case 1

A male aged 28 was observed in the oral diagnosis department with a primary concern of expansion in the left anterior maxilla region from the distal surface of the left maxillary central incisor to the mesial surface of the left maxillary canine and with pain in associated teeth since about a month. The mass progressively expanded to its present size. He had a history of trauma in the region at around the same time subsequent to which he noticed the swelling. His medical history included treatment for tuberculosis for 2 years, prior to which he also had the habit of gutka consumption.

On extraoral assessment, a facial swelling was visible with a diffuse, firm, non-sore, enlargement of the left malar area along with an obliteration of the nasolabial fold on the left side of the face and elevation of the left ala of the nose. The overlying skin was of normal color and temperature. The size of the swelling was about 4*2 cm.

**Figure 1:**
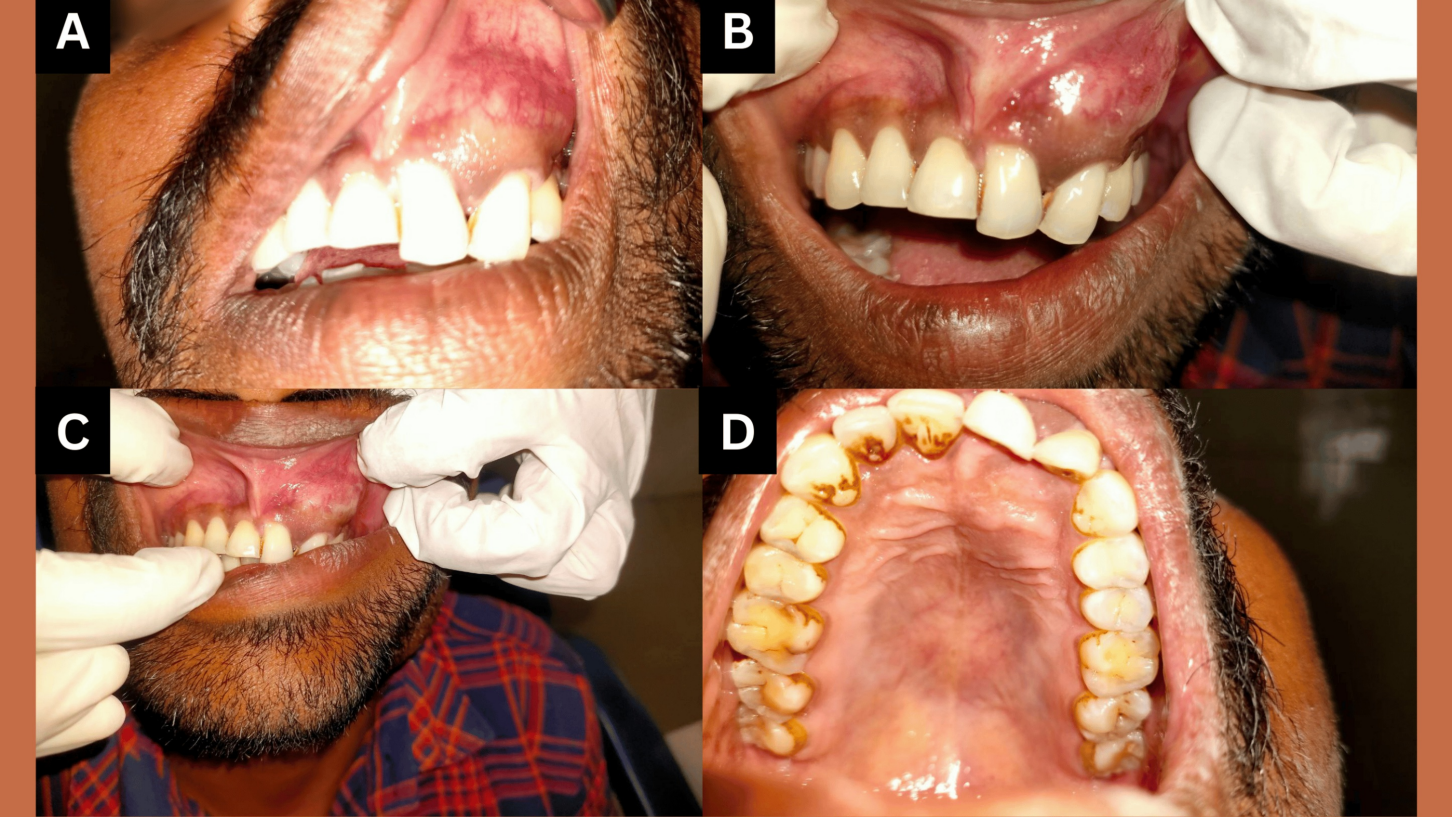
Clinical photographs of Case 1 (A) Intraoral swelling; (B,C) Maxillary anterior vestibule obliteration by swelling; (D) Palatal swelling in the left maxillary anterior region.

Intraorally, a solid, well-confined, isolated swelling was present covering the area from the gingiva to alveolar mucosa of left anterior teeth from the distal region of maxillary left central incisor to the mesial area of the left maxillary canine and extending to the vestibule above. As a result of enlargement and lateral increase in the buccal cortical plate of the region, there was a resultant obliteration of the vestibule in the region (Figure 1A-1C). Palatally, a mild swelling was noted in the area of left maxillary incisors (Figure 1D). The overlying mucosa was observed to be of normal color. The swelling was 2.5*2 cm in size, firm to palpation with a smooth overlying surface.

**Figure 2:**
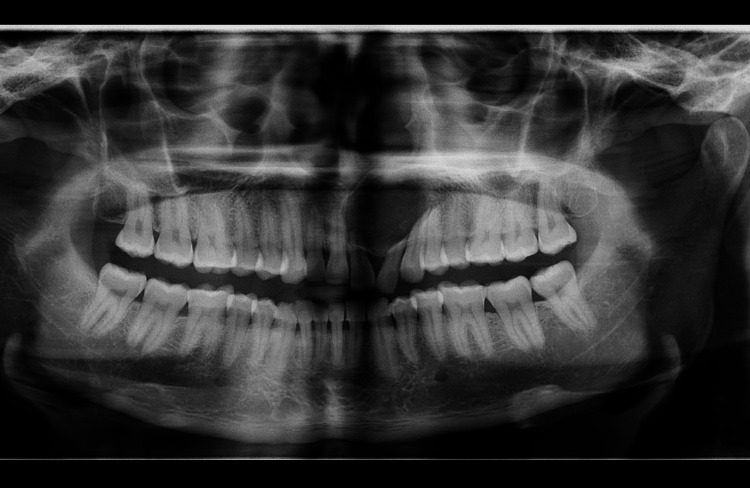
Case 1: OPG showing well-demarcated radiolucency in left maxillary anterior region with Incisor roots deflection and resorption OPG: Orthopantomogram

On fine needle aspiration, a straw-colored fluid was aspirated. The panoramic radiograph exposed a huge, distinct unilocular, round-to-ovoid radiolucency existing in the maxilla, extending from the left maxillary central incisor to the mesial surface of the left maxillary first premolar and it also showed, radicular resorption of the adjacent teeth (Figure 2).

A clinical diagnosis of COC was done based on the clinical presentation, patient history, and radiographic picture, and AOT (adenomatoid odontogenic cyst) and CEOT (calcifying epithelial odontogenic cyst) were the differential diagnostic pathologies.

**Figure 3:**
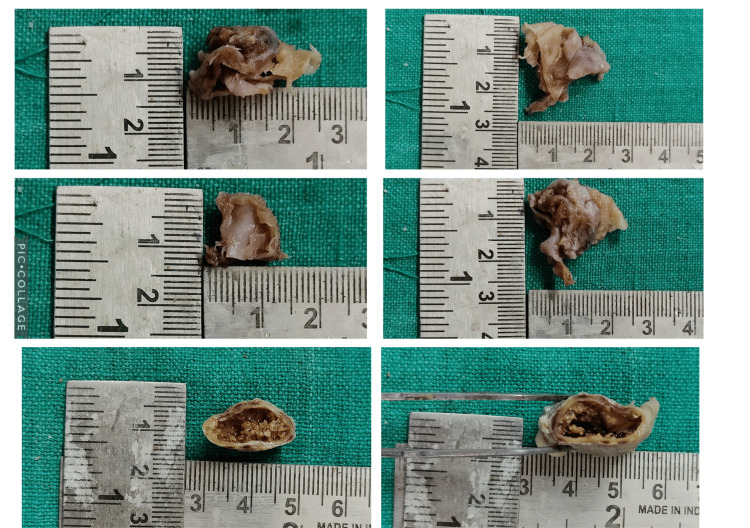
Case 1: Gross tissue specimen images with dimensions

Under local anesthesia, the tumor was surgically excised and the bone was curetted. Primary closure was attained. The surgical specimen was sent for analysis by pathologists. Grossing revealed that the surgical specimen was a single, piece of soft tissue with a corrugated surface that was grey-brown in color and was firm to the touch (Figure 3).

From the surgical sample, representative tissue was removed, fixed in formalin, and then processed according to standard protocol, and stained with hematoxylin and eosin. A few days after the cyst was enucleated, the maxillary left lateral incisor was also removed. 

**Figure 4:**
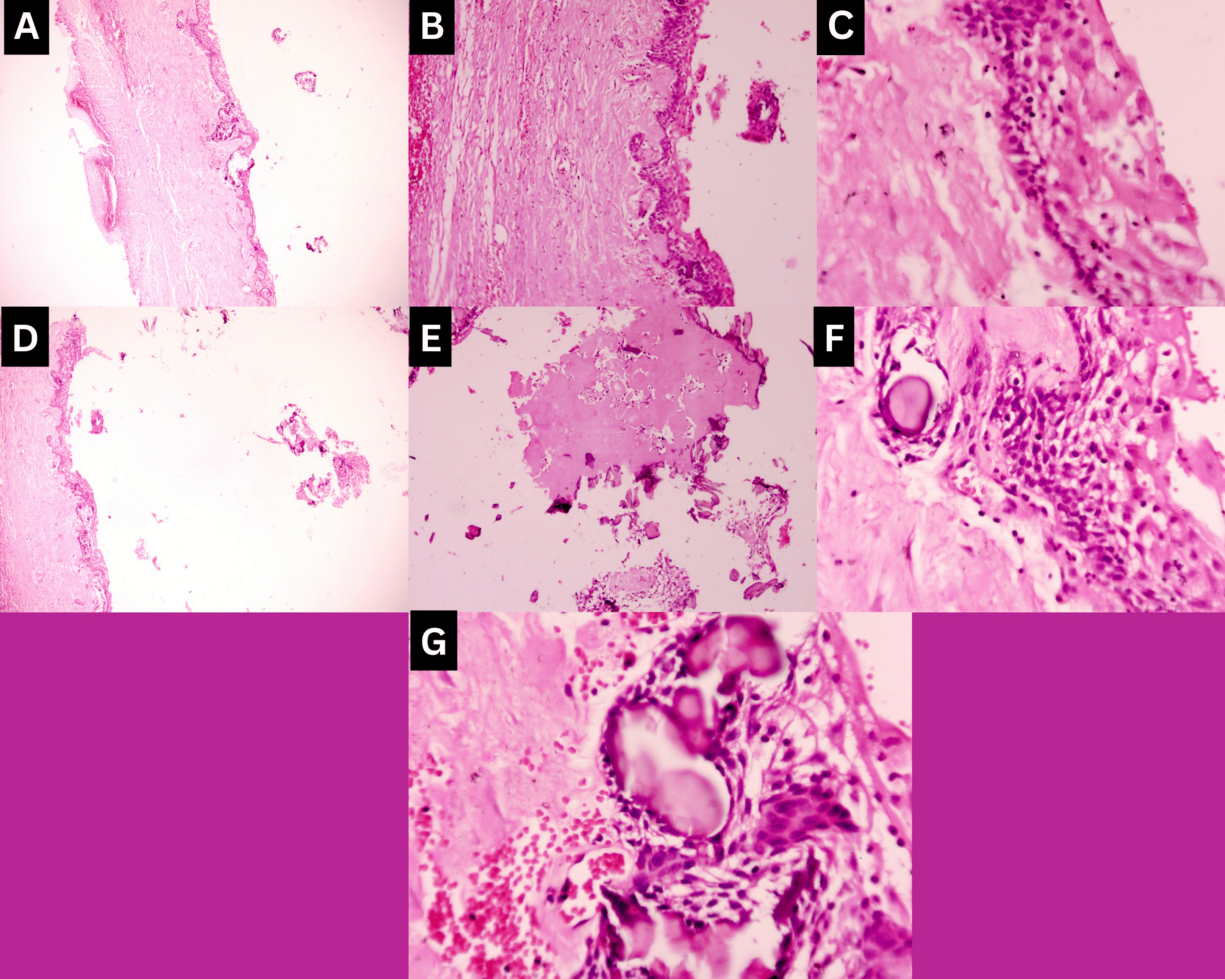
Histopathological images of Case 1 (A-C) 4X, 10X, 40X magnification of cystic lining and capsule; (D-G) Eosinophilic amorphous material, ghost cells, and calcifications.

Histopathological analysis of the excisional biopsy specimen of the patient supported the clinical diagnosis of a COC, as it showed a cystic lesion with, a lining epithelium of non-keratinised odontogenic epithelium in which the basal cells were cuboidal to columnar with, palisading, hyperchromatic nucleus similar to, the cells with ameloblastic differentiation and the epithelial lining was of variable thickness (Figure 4A-4C). The suprabasal epithelial cells were morphologically, similar to stellate reticulum cells of the enamel organ and in many foci showed pale, eosinophilic, anucleate ghost cells. These ghost cells were also seen to undergo keratinisation and/or calcification materializing sheets of dense homogenous eosinophilic matter and basophilic dystrophic calcifications (Figure 4D-4G). The connective tissue capsule was fibro-cellular with the presence of pale eosinophilic, irregular-shaped calcifications suggesting dentinoid material.

Followup of the patient has been uneventful so far for about one year.

Case 2

A female aged 25 was examined clinically in the oral medicine outpatient clinic for her presenting complaint of a gradually increasing lumpy, mass with pain in associated teeth since about one year in the left maxillary area from the distal surface of the maxillary left canine to the mesial surface of left maxillary first premolar and apical region of the left maxillary second premolar. She had an extra-oral swelling of the mid-face region at the time of presentation.

Her past medical, dental as well as family history was non-significant. Her extra-oral swelling was about 5*6 cm in the greatest dimensions. It extended from the left ala of the nose to a line parallel to the zygomatic arch medio-laterally and from the corner of the mouth to a line drawn parallel to the lower border of the zygomatic arch superior-inferiorly. However, the color of the swelling was the same as the normal skin. 

**Figure 5:**
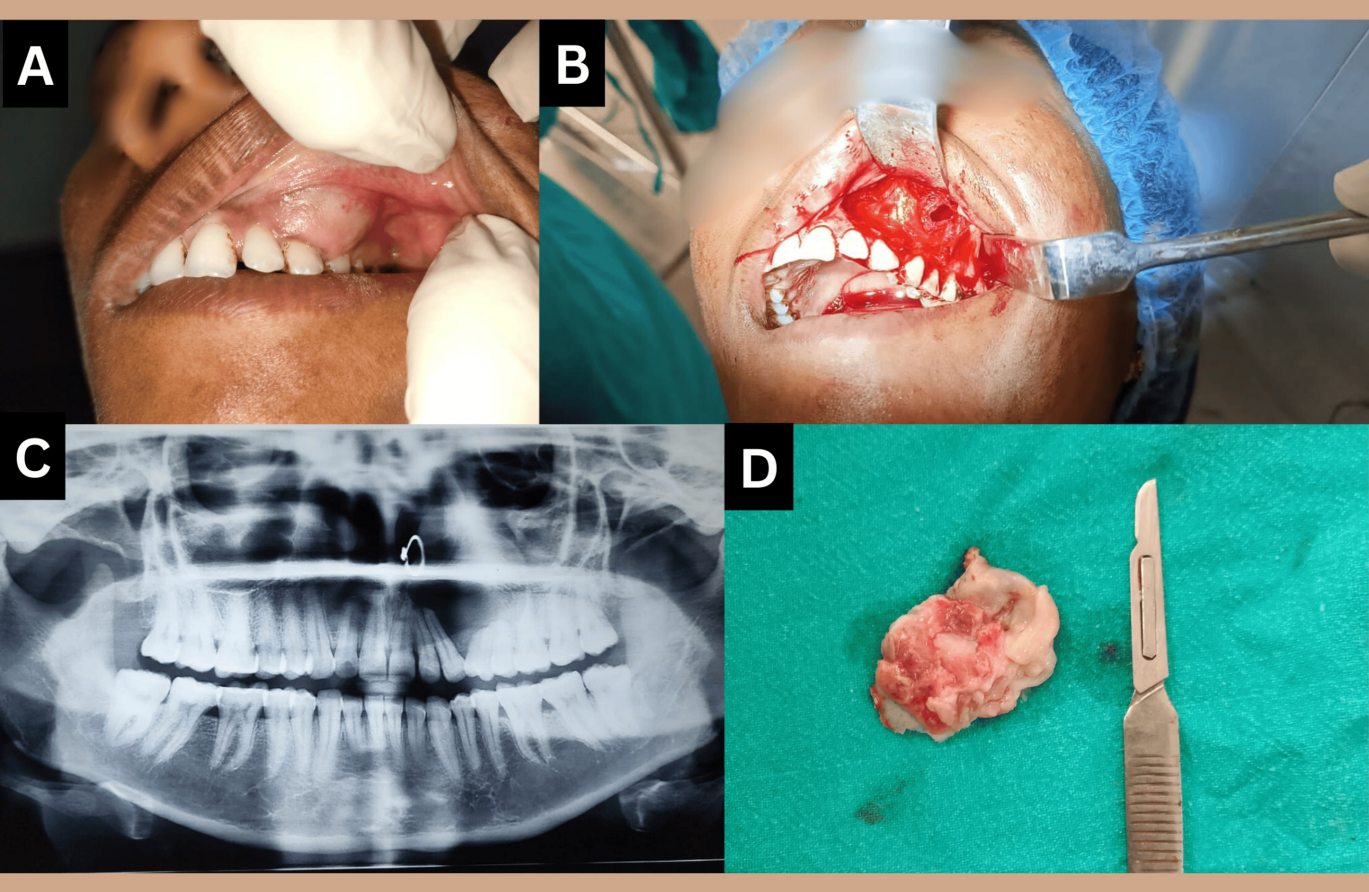
Case 1: Clinical and radiological images (A) Clinical image of swelling in maxillary left canine-premolar region vestibule; (B) Surgical image; (C) X-ray image; (D) Surgical specimen

Intra-orally, a swelling was present in the gingiva and alveolo-buccal sulcus associated with the left maxillary canine and the first premolar, and it obliterated the vestibule of the region. The swelling had diffuse margins, with an outgrowth of the buccal cortical plate of the region. The overlying mucosa was of normal gingival color. The swelling had a firm consistency with a smooth overlying surface, and crepitation was felt but the lesion was non-tender on palpation. The size of the lesion was approximately 4*3 cm (Figure 5A, 5B).

On fine needle aspiration, a straw-coloured fluid with pus was found. A panoramic radiograph exposed a large well-defined unilocular, round to ovoid radiolucency present in the maxilla, extending from the left canine to second premolar, and it had caused a divergence of roots of canine and first premolar with resorption of first premolar roots (Figure 5C)

A clinical diagnosis of COC was given with differential diagnosis of, infected dentigerous cyst, and CEOT. Surgical enucleation of the lesion and bone curettage were performed under local anaesthesia subsequent to root canal treatment of the involved teeth (Figure 5D). Root canal treatment of involved teeth was done prior to surgical removal of cyst to rule out any infective focus.

**Figure 6:**
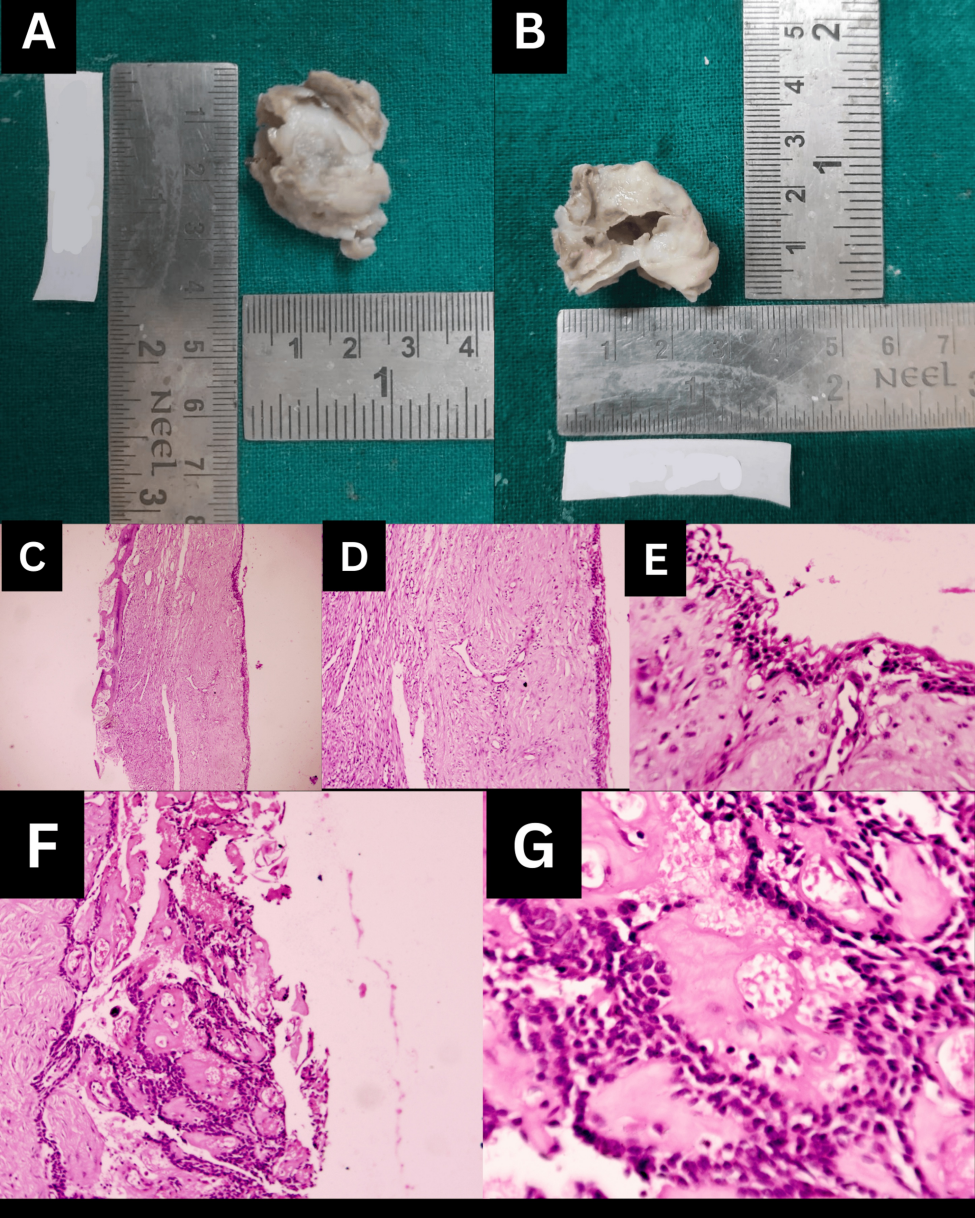
Case 2: Gross tissue specimen (A-B) Gross tissue specimen; (C-E) 4X, 10X, and 40X magnification of cystic lining; (F-G) 10X and 40X magnification of aberrant keratinization-like ghost cell areas surrounded by ameloblast-like epithelial  cells

The pathological defect was closed with primary closure. The surgical sample was sent for histopathological assessment. On grossing, the surgical specimen consisted of single, whitish-grey colored, firm, saclike tissue that measured 3*2*0.6 cm in its greatest dimension. It was firm to palpation with a corrugated surface (Figure 6A, 6B).

Representative tissue samples taken from the surgical sample were fixed in formalin and routinely processed. Then they were stained with haematoxylin and eosin (H&E) for histopathological reporting.

H&E stained tissue sections of the excisional biopsy specimen of the patient supported the diagnosis of a COC as - it showed a cystic lesion which, in 4X, 10X, and 40X magnification showed a lining epithelium of non-keratinised odontogenic epithelium in which the basal layer of cells was cuboidal to columnar with palisading, hyperchromatic nucleus similar to ameloblastic differentiation and the epithelial lining was of variable thickness(Figure 6C-6E). In 10X and 40X magnification, the suprabasal epithelial cells resembled the stellate reticulum of the enamel organ and in many foci showed pale, eosinophilic, anucleate ghost cells (Figure 6F, 6G).

**Figure 7:**
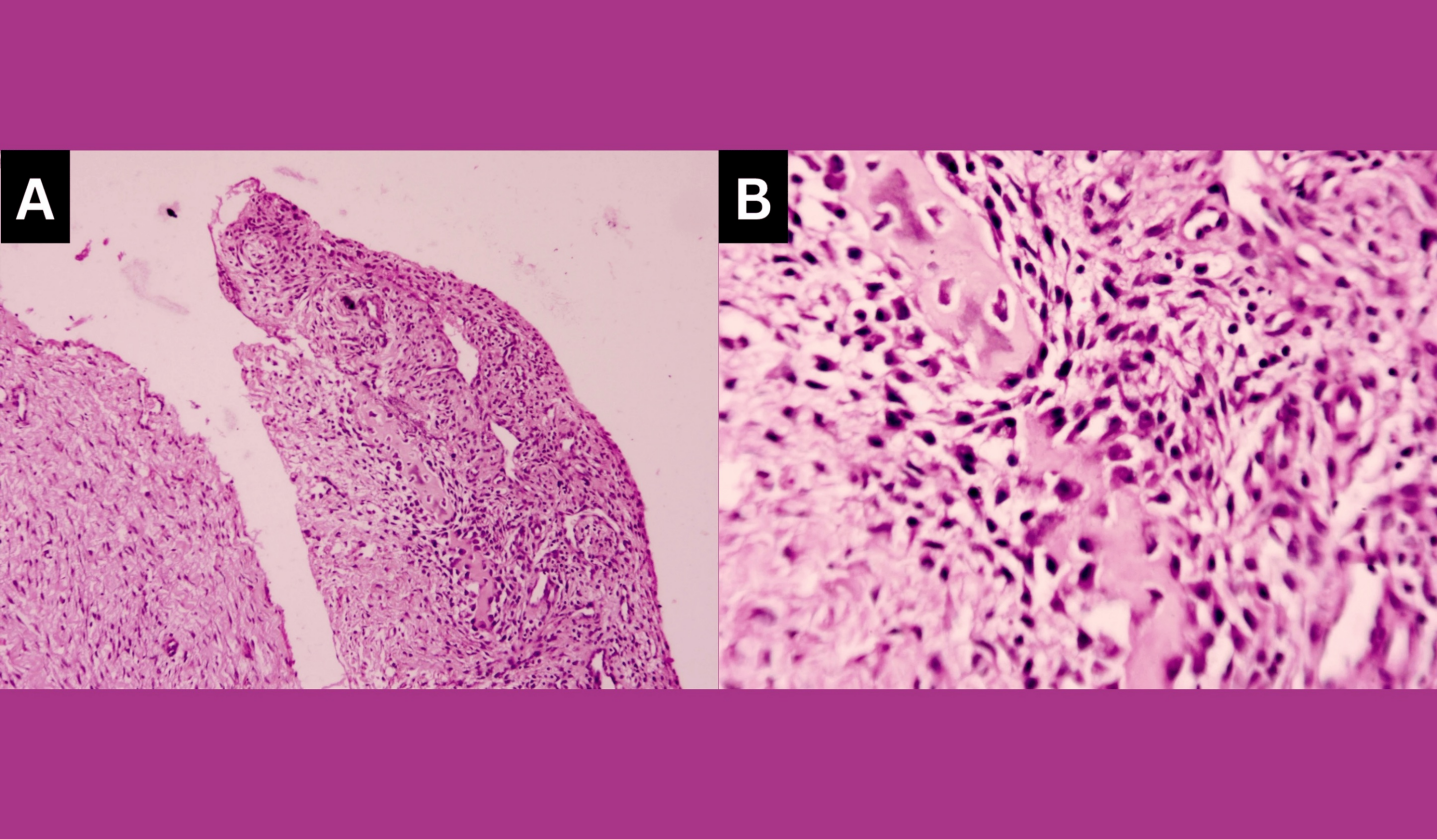
Case 2: Histopathology (A-B) 10X and 40X magnification of dentinoid material surrounded by stellate reticulum-like cells with plump connective tissue cells.

The connective tissue capsule was fibro-cellular with the presence of pale eosinophilic, irregular-shaped calcifications suggesting, dentinoid material surrounded by stellate reticulum-like cells and, with a hyper-cellular connective tissue stroma containing plump fibroblasts was seen surrounding these foci. These ghost cells were also seen to undergo keratinisation and/or calcification forming sheets of dense homogenous eosinophilic matter and basophilic dystrophic calcifications (Figure 7). An area of connective tissue capsule showed the presence of giant cells also. In another area, moderate inflammatory cell infiltration was seen.

Post-operative follow-up has been uneventful for more than six months post-surgery.

Table 1 compares the findings of both cases.

**Table 1:** Comparative table of findings of both cases

Type of Observation	Case 1	Case 2
Sex	Male	Female
Age	Second decade	Second decade
Duration of lesion	1-2 months	11-12 months
Jaw affected	Maxilla	Maxilla
Site	Incisor-Canine region	Canine-Premolar region
Chief complaint	Swelling with tenderness in associated teeth	Swelling with tenderness in associated teeth
Size of lesion	2.5*2 cm	4*3 cm
Xray findings	Divergence of roots of lateral incisor, canine, and first premolar with resorption of roots of lateral incisor and canine	Divergence of roots of canine and first premolar with resorption of roots of the first premolar
Cystic fluid	Straw colored	Straw colored
Treatment	Cyst enucleation	Cyst enucleation
Histopathology	1. Cystic lining of ameloblast-like basal cells with overlying stellate reticulum-like cells. 2. Presence of ghost cells and aberrant keratinization in lumen 3. Calcifications in cystic capsule 4. Cyst capsule - Predominantly fibrous	1. Cystic lining of ameloblast-like basal cells with overlying stellate reticulum-like cells. 2. Presence of like cells stellate reticulum dentinoid material surrounded by stellate reticulum-like cells 3. Presence of ghost cells in lining 4. Presence of Giant cells in cyst capsule 5. Cyst capsule - Predominantly cellular with plump fibroblasts

## Discussion

True bone cysts can develop in the maxilla and mandible because they have embryonic epithelial resting cells in them. The majority of them are odontogenic.

When the World Health Organization (WHO) classified the COC as part of its "Histological Typing of Odontogenic Tumors, Jaw Cysts, and Allied Lesions" classification in 1971, the COC became well-known throughout the world. Even though COC appears as a cystic lesion, all COCs were classed as neoplastic lesions according to the WHO's monistic definition. There has been debate and misunderstanding over the nomenclature and categorization of this lesion because of its variability for a long time. Its classification remains up for discussion. The WHO classified it as an odontogenic tumor in 1992 [5].

It is usual for oral and maxillofacial surgeons to encounter asymptomatic periapical diseases, and calcifying odontogenic cyst, a central (Intrabony) odontogenic cystic pathology, is one such lesion. Except for being radiographically radiolucent with isolated patches of radiopacity, this cystic lesion is asymptomatic. It is also common for 15-25% of these lesions to develop extraosseously in the gingiva. The pathology was initially identified in 1932. Later, it was defined and described as a separate lesion in 1962, citing the significant number of "ghost cells" in the lesion as well as the histological similarity between the calcifying odontogenic cyst (COC) and the cutaneous calcifying epithelioma of Malherbe. The term "Gorlin cyst" is commonly used as its eponym. However, the origins, pathophysiology, and variations in the histopathology of COC have evoked considerable discussion in the literature [5,6].

The etiopathogenesis of the cyst formation is rests of Serres, present in the oral connective tissues or alveolar bone. This indicates that COC are of primordial origin and are not formed subsequent to tooth formation and therefore, are not correlated with the crown of an unerupted tooth. It is often seen that COC occurs with another odontogenic lesion like odontoma, ameloblastoma, or adenomatoid odontogenic tumor. Neither of our cases showed any other odontogenic lesion with COC [5].

According to a publication by Menditti D et al (2020), beta-catenin mutations have been found in the cytoplasm and nuclei of a number of COCs [2]. The authors speculate that these mutations may play a significant role in the pathogenesis of these lesions because numerous studies have established a connection between the activation of the Wnt/Beta-catenin pathway, the establishment of ghost cells, and their calcified structures. In order to induce tooth creation, the Wnt gene signaling system is closely linked to the formation of dental hard tissue. Consequently, it supports the theory that the Wnt signaling pathway is altered as a result of this mutation and that this is what causes cancer in COC. Therefore, the pathophysiology of benign odontogenic ghost cell lesions such as COC probably involves aberrant signaling in the odontogenic epithelium regulated by beta-catenin. Some researchers have also noted CTNNB1 point mutations in COC [2-6].

There is an almost even gender distribution with a negligible difference in gender distribution. Asians have a higher frequency in younger age groups, particularly, in the second and third decades and over 70% occur in these decades, compared to just around 53% in the corresponding decades for White people. Our two instances, one male and the other female, were both in their second decade. Lesions in Asians indicate a propensity for the maxilla (65%), while in Whites, the mandible (62%), is the preferred location. It demonstrates a preference for the anterior region of the jaws as a place of occurrence. Both our cases had occurred in the maxillary anterior-premolar region. A few cases have been seen to have crossed the midline in the mandible, however, this is less common in the maxilla. 

Intra-osseous lesions may generate a hard bony extension and may be fairly general. A persistent lesion-associated complaint usually is swelling in the affected region in a large number of the reported cases. In some cases, displacement of the teeth has been explained and when COC is associated with apices of teeth, it shows a high rate of root resorption. In our cases, the swelling was the chief complaint with lesion-associated teeth having tenderness and displacement [6].

Although the lesion may occasionally be multilocular, radiographically, intraosseous lesions are mostly unilocular, well-defined to poorly defined radiolucency. There is a chance that radiopacities - either irregular calcifications or tooth-like densities - will be present inside the radiolucency. Teeth displacement can be appreciated as a common occurrence radiologically. Some researchers have noted that a common and significant radiological result of COC was root resorption of neighboring teeth's roots [6, 7].

Root resorption and neighboring tooth displacement were also observed in our patients.

On account of being rare, the pathophysiology of COC is yet unknown. Its clinical and radiographic symptoms are not pathognomonic but rather its histo-pathological aspects are what really define it.

The basal layer of the epithelial lining of the COC exhibits histological findings that resemble ameloblasts, appearing columnar or cuboid in shape. The suprabasal layers of cystic lining frequently display a cellular structure like that of a stellate reticulum, as observed in the enamel organ. This lesion is notable for the anucleated, mildly eosinophilic cells that are present and these cells are known as "ghost cells." There is also dysplastic dentin in the fibrous capsule [7].

Ghost cells can arise from a number of different processes, according to research findings. These include aberrant keratinisation, coagulative necrosis, metaplastic transformation, abortive formation of enamel matrix, local hypoxia and degeneration, and/or accumulation of hard keratin [8].

Clinical differential diagnosis includes common non-neoplastic gingival lesions odontogenic keratocyst, adenomatoid odontogenic tumor, and other extra-osseous odontogenic tumors. Benign radiolucent lesions such as ameloblastoma, adenomatoid odontogenic tumor, dentigerous cyst, odontogenic keratocyst, periapical cyst, ameloblastic fibro-odontoma, and calcifying epithelial odontogenic tumor are among the lesions that can be considered in radiological differential diagnosis [9].

The presence of ghost cells in the cyst cavity and/or lining epithelium is the most noticeable characteristic of COC. The presence of ghost cells is characterized by the suprabasal cells being widely separated by intercellular oedema and the surrounding epithelium being convoluted. Morphologically, ghost cells are eosinophilic epithelial cells that are swollen, inflated, ovoid or elongated, ellipsoid, and frequently have well-defined to blurred cell outlines. Cell groups seem merged when cell outlines become blurry. Ghost cells can occasionally have nuclear remains at different stages of degeneration; as a result, the nucleus is only partially defined and has evidence of chromatin. The ghost cells show a preference for calcification and are also an example of an aberrant keratinization. According to Praetorious et al (1981) [10], ghost cells display the same histological outcomes as keratin when stained with rhodamine B and elicit a yellow fluorescence [8-10].

In both of our cases, the cystic lining was of ameloblast-like hyperchromatic and polarized basal cells and suprabasal stellate reticulum-like cells with the presence of ghost cells with or without calcification among them.

According to the most recent WHO categorization from 2017, COC is classified as an intraosseous and extraosseous developing cyst. Simple unicystic, unicystic odontoma-producing, and unicystic with ameloblastomatous growth are the three forms identified in the cystic variation [9-11].

A similarity of clinical and radiological presentation of COC with other odontogenic lesions that are unilocular commonly and multilocular less commonly coupled with asymptomatic presentation warrants a thorough pathological examination for confirmation of diagnosis which, in turn, is essential as COC has an aggressive behaviour and a potential for the recurrence [12,13].

There is an extremely low chance of calcifying odontogenic cysts recurring and just eight such cases have been reported in the literature. Additionally, the malignant counterpart of COC, ghost cell odontogenic carcinoma has also been reported to occur either de novo or as a recurrence of COC and Motosugi U et al had reported it to have occurred in 3/122 cases studied by them. They also stated that histological and immunohistochemical evaluation was essential to its diagnosis [14].

**Table 2:** Immunohistochemistry studies done to analyze the nature of ghost cells with their results COC: calcifying odontogenic cysts; CK-19: cytokeratin 19; MMP-20: Matrix metalloproteinase-20; PCNA: proliferating cell nuclear antigen; COX-2: cyclooxygenase 2; CD-1: cyclin D1

Authors & Year	Cells studied	Immunohistochemistry results
Takata et al (2000) [15]	Compared nature of ghost cells in COC and calcifying epitheliomas of Malherbe	COC Ghost cells: +ve for enamel-related proteins (enamelysin/MMP-20)
Abiko et al (2001) [16]	Immunoreactivity of Ghost cells of COC to Amelogenin	+VE reaction to amelogenin by ghost cells
Yoshida et al(2001) [17]	Immunoreactivity of Ghost cells of COC to Amelogenin, CK-19, and bcl-2	+ve reaction to amelogenin in ghost cells of all COC samples, +VE reaction to CK-19 and bcl-2 in basal cells of the epithelial lining and -ve in ghost cells
Fregnani et al (2003) [18]	Epithelial lining of COC cases	Ghost cells expressed only cytokeratins AE1/AE3 and 34βE12, cytokeratins 14 and AE1/AE3 were expressed in the basal cells of the epithelial linings of all cases, +ve bcl-2 in basal and suprabasal cells but negative in ghost cells
Kusuma et al (2005) [19]	Studied immunoreactivity of ghost cells to human hair proteins	+ve reaction only of ghost cells of COC indicated these cells to be related to hair differentiation and also +ve for phosphothreonine found in hard alpha keratins
Saghafi et al. (2010) [20]	Immunoreactivity to p53 and PCNA of basal/suprabasal cells and ghost cells of COC	a) Strong +VE reaction to p53 in basal/suprabasal cells -ve reaction to p53 in ghost cells b) Moderate +ve reaction to PCNA in basal/suprabasal cells -ve reaction to PCNA in ghost cells
Arruda J A (2018) [21]	Immunoreactivity to COX-2 and CD-1 of COC epithelial lining	Moderate +ve reaction of basal and suprabasal cells to CD-1, negative reaction of epithelial lining to COX-2
Urs AB et al (2020) [22]	Immunoreactivity to CK-6, CK-19, and amelogenin of basal/suprabasal cells and ghost cells of COC	Strong +ve reaction to amelogenin in ghost cells of all COC samples, moderate +ve reaction to CK-6 in ghost cells of all COC samples, +ve reaction to CK-19 in basal/suprabasal cells, -ve reaction to CK-19 in ghost cells
Araujo ET AL (2020) [23] Mulvihill C et al (2020) [4]	Immunoreactivity to β-Catenin of basal/suprabasal cells and ghost cells of COC	Weak +vity in basal/suprabasal cells; strong immunopositivity in cells surrounding ghost cells
Blanca Urzúa et al (2021) [24] Mulvihill C et al (2020) [4]	Immunoreactivity to amelogenin, cytokeratin AE1/AE3 (CKAE1/AE3), and cytokeratin 14 (CK14) of basal/suprabasal cells and ghost cells of COC	Amelogenin +ve in ghost cells, CKAE1/AE3 +ve in both basal and ghost cells, CK-14 +ve in basal cells and -ve in ghost cells

In a continuous effort to give it a distinct status, its clinical behaviour was evaluated by some researchers, and one of the methods to do so was to adjudge its epithelial lining's proliferative capacity. The proliferative markers' immunostaining of COC lining epithelium suggested that it had a low proliferative capacity and aggressiveness, similar to a cystic lesion and not a neoplasm [20].

Ultrastructurally, the tonofilaments of ghost cells differ from that of normal squamous cells, in that they are an aberrant type or defective type of haphazardly arranged tonofilaments that fail to aggregate as bundles of tonofibrils and also lack the presence of keratohyaline granules [20,21].

In the 2017 WHO classification, COCs were recategorized as cystic pathologies and additionally. It was noted that, though ghost cells may be seen in various other pathologies, for COC, the presence of these cells was pathognomic and mandatory for confirmatory histopathological diagnosis of COC [22].

The WHO recently published a classification for odontogenic and maxillofacial bone tumors. This classification suggests a minor modification to the diagnostic criteria for COC, whereby the presence of ghost cells that have the potential to calcify is sufficient for making a diagnosis, while the presence of the ameloblastic epithelial lining is not a requirement. Furthermore, odontoma with COC is no longer regarded as a distinct subgroup of COC [23].

The biological behaviour of COC is also reasserted by the presence of amelogenin protein in the aprismatic, amorphous, calcified matrices of epithelial origin in COC, suggesting that the cells secreting it are highly differentiated odontogenic epithelium simulating ameloblast function, and hence, low proliferation capacity and also less aggressive behaviour [24].

Thus, calcifying odontogenic cyst (COC) is a distinct pathology with separate pathogenesis, clinical behaviour, and distinct histopathology along with its epithelial lining cells having an inherent capacity to secrete enamel protein-like substance and attempt initiation of dental hard tissue formation or aberrant keratinization or both [25].

The capacity to initiate the formation of dental hard tissues appears to be an inherent property of epithelial lining cells of COC. Also, ghost cells are believed to be a consequence of other processes like coagulative necrosis followed by dystrophic calcification, or a form of normal or abnormal keratinization of the odontogenic epithelium. 

The conservative surgical enucleation of the whole cyst is the first step in treating COC, and it is followed by curettage and follow-up. 

## Conclusions

The two cases presented in this paper have been seen in different sexes but had similar clinical, radiological, and histological presentations. This cystic pathology has always been debated due to its association with various other odontogenic pathologies as well as its variable clinical and radiological presentation and tendency for resorption of teeth roots in the vicinity. Another reason is the debatable nature and origin of the histological features of ghost cells.

The limitations of our study are that we could only report two cases of COC and we could not evaluate the nature of ghost cells in our cases by immunohistochemistry to throw more light on their origin and occurrence in COC. However, with more cases being published, further clarity in these contexts is likely along with further analysis of their data in the literature.
